# Genetic diversity and relationship of Indian cattle inferred from microsatellite and mitochondrial DNA markers

**DOI:** 10.1186/s12863-015-0221-0

**Published:** 2015-06-30

**Authors:** Rekha Sharma, Amit Kishore, Manishi Mukesh, Sonika Ahlawat, Avishek Maitra, Ashwni Kumar Pandey, Madhu Sudan Tantia

**Affiliations:** Core lab (Network Project Unit), National Bureau of Animal Genetic Resources, G T Road, Karnal, 132001 Haryana India

**Keywords:** Conservation, Diversity, Genetic relationship, Indian cattle, Microsatellite markers, Mitochondrial DNA, Population structure

## Abstract

**Background:**

Indian agriculture is an economic symbiosis of crop and livestock production with cattle as the foundation. Sadly, the population of indigenous cattle (*Bos indicus*) is declining (8.94 % in last decade) and needs immediate scientific management. Genetic characterization is the first step in the development of proper management strategies for preserving genetic diversity and preventing undesirable loss of alleles. Thus, in this study we investigated genetic diversity and relationship among eleven Indian cattle breeds using 21 microsatellite markers and mitochondrial D loop sequence.

**Results:**

The analysis of autosomal DNA was performed on 508 cattle which exhibited sufficient genetic diversity across all the breeds. Estimates of mean allele number and observed heterozygosity across all loci and population were 8.784 ± 0.25 and 0.653 ± 0.014, respectively. Differences among breeds accounted for 13.3 % of total genetic variability. Despite high genetic diversity, significant inbreeding was also observed within eight populations. Genetic distances and cluster analysis showed a close relationship between breeds according to proximity in geographic distribution. The genetic distance, STRUCTURE and Principal Coordinate Analysis concluded that the Southern Indian Ongole cattle are the most distinct among the investigated cattle populations. Sequencing of hypervariable mitochondrial DNA region on a subset of 170 cattle revealed sixty haplotypes with haplotypic diversity of 0.90240, nucleotide diversity of 0.02688 and average number of nucleotide differences as 6.07407. Two major star clusters for haplotypes indicated population expansion for Indian cattle.

**Conclusions:**

Nuclear and mitochondrial genomes show a similar pattern of genetic variability and genetic differentiation. Various analyses concluded that the Southern breed ‘Ongole’ was distinct from breeds of Northern/ Central India. Overall these results provide basic information about genetic diversity and structure of Indian cattle which should have implications for management and conservation of indicine cattle diversity.

**Electronic supplementary material:**

The online version of this article (doi:10.1186/s12863-015-0221-0) contains supplementary material, which is available to authorized users.

## Background

India is home to the largest cattle population (13.1 % of world’s cattle population) in the world which constitutes 37.3 % of its total livestock [1]. Indian zebu cattle (*Bos indicus*) evolved over centuries under low levels of selection followed in traditional animal husbandry. As a result, Indian cattle adapted to harsh native environment, resistance to tropical diseases and external parasites and sustenance on low quality roughages and grasses. A large and divergent range of agro-ecological zones in India have helped to develop number of cattle populations. The state of world’s animal genetic resources, SoW-AnGR listed a total of 60 local, eight regional trans-boundary and seven international trans-boundary cattle breeds from India [2]. Among these very few are maintained for milk production (Sahiwal, Gir, Rathi and Sindhi), some are dual-purpose breeds (Deoni, Hariana, Kankrej and Tharparkar) while the rest are draft breeds, maintained by farmers for producing bullocks. With the modernization of agriculture and sub-division of land holdings, bullock power in Indian agriculture is losing its importance. Thus, many of the draft breeds are under severe neglect resulting in continuous decline of indigenous cattle population [1]. In addition, introduction of highly productive breeds and demographic pressure are also contributing to the loss of valuable traits or decrease in population of local breeds.

Genetic characterization of breeds allows evaluation of genetic variability, a fundamental element in working out breeding strategies and genetic conservation plans. Molecular markers have revolutionized our ability to characterize genetic variation and rationalize genetic selection [3]. Markers have been comprehensively exploited to access genetic variability as they contribute information on every region of the genome, regardless of the level of gene expression. Employment of microsatellite markers is one of the most powerful means for studying the genetic diversity, calculation of genetic distances, detection of bottlenecks and admixture because of high degree of polymorphism, random distribution across the genome, codominance and neutrality with respect to selection [4]. Mitochondrial DNA (mtDNA) is also considered to be a good tool for genetic diversity and evolutionary studies due to near-neutrality, maternal inheritance and clock-like nature of its substitution rate [5]. The Displacement region (D-loop) is proven to be a particularly useful genetic marker because it evolves much rapidly than the coding region of the mtDNA [6]. Direct comparisons between mtDNA and microsatellite loci can be very informative for population diversity and genetic structure, as evolutionary forces affect each class of marker differently [7].

Considering the importance of cattle in Indian agriculture, few efforts have been made to evaluate the genetic diversity and relationship in Indian cattle using microsatellite markers [8–12]. However, ecomprehensive knowledge of the breed characteristics, including within-and between-breed genetic diversity which will result in complete representation possible of biological diversity is required to facilitate effective management. Thus, a deeper knowledge of the genetic diversity and population structure of Indian cattle can provide a rational basis for the need of conservation and possible use of native breeds as genetic resources to meet potential future demand of adaptation to changing environment or production needs. Therefore, the present investigation was undertaken to quantify the genetic diversity and relationship between eleven cattle breeds of India.

The objectives of this study were to use microsatellite markers and mitochondrial DNA control region polymorphisms to characterize the within-breed genetic diversity, to establish breed relationships and to assess their population structure. The use of molecular information supplied by nuclear and mtDNA markers is aimed to provide a rational basis for suitable strategies of management and conservation.

## Method

### Sample collection and DNA extraction

**Fig. 1:**
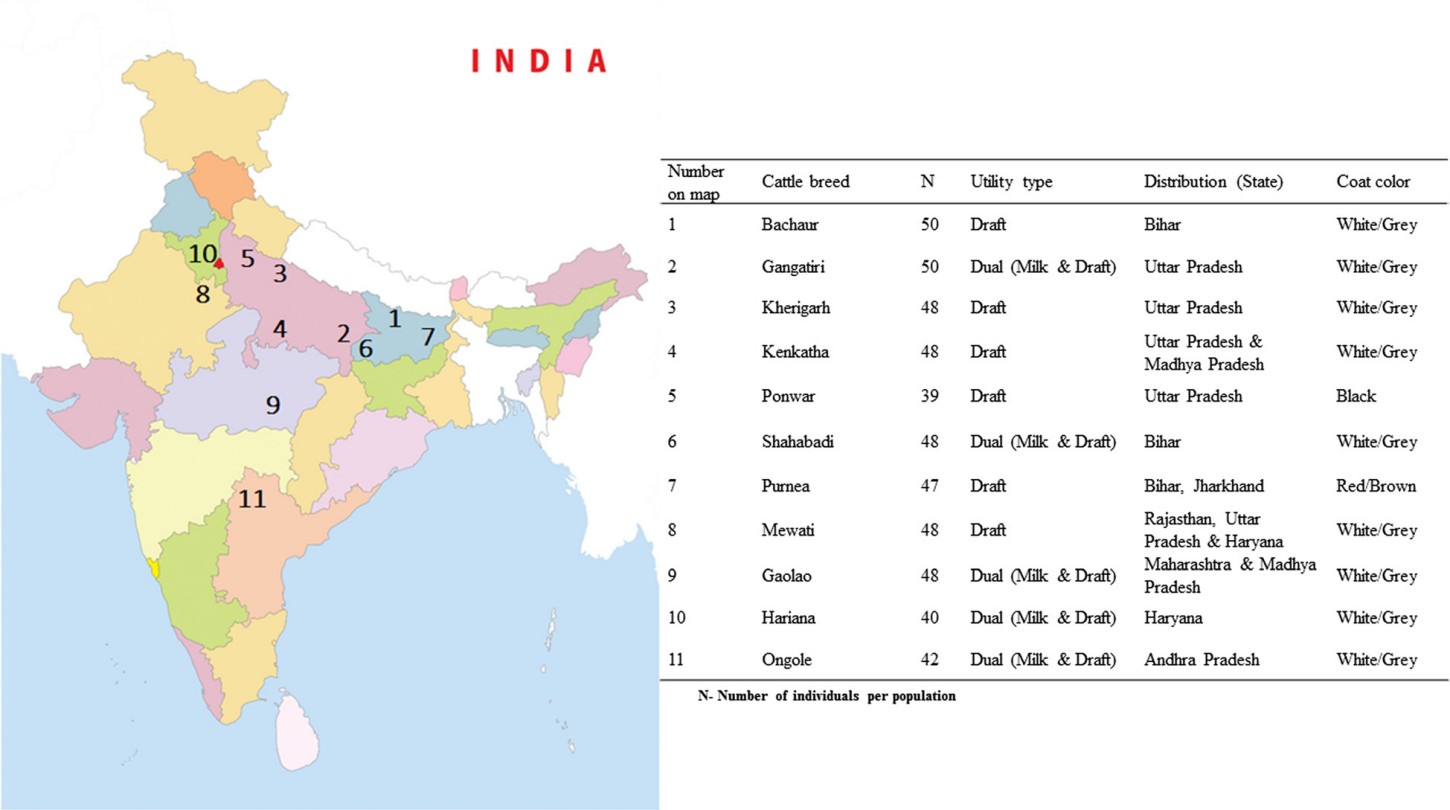
Geographic distribution and characteristics of Indian cattle populations analyzed in the present study

No animal experiments were performed in this study, and, therefore, approval from the ethics committee was not required. Blood samples were collected with the help of veterinary doctors from respective State Animal Husbandry Department. In total, 508 animals from 11 different cattle breeds (Bachaur-50, Gangatiri-50, Kherigarh-48, Kenkatha-48, Ponwar-39, Shahabadi-48, Purnea-47, Mewati-48, Gaolao-48, Hariana-40 and Ongole-42) were sampled from Northern, Central and Southern India (Fig. 1). Samples of the populations included in this study represented animals of the original autochthonous phenotype. To ensure random sampling, animals were selected from different villages of habitat while avoiding closely related individuals on the basis of detailed interview with owners. Blood samples were collected from jugular vein in 10 ml vacuitainer tubes with EDTA as anticoagulant and were stored at–20 °C until DNA extraction. Genomic DNA was isolated from blood using Phenol-chloroform method as described by Sambrook and Russel [13].

### Microsatellite polymorphism

DNA samples were amplified by PCR in correspondence with the selected panel of 21 bovine specific loci. The loci were chosen, according to ISAG/FAO recommendation aiming to analyze high polymorphic markers spread all over the genome and with the ability to co-amplify in PCR reactions [14]. The fluorochrome labeled (FAM, NED, PET& VIC) primers were synthesized by Applied Biosystems (Table 1). For amplification, 50-100 ng of genomic DNA was added to a reaction mixture containing 50 pMol of primer-forward and reverse, 200 μM of each dNTPs, 1.5 mM of MgCl_2_ and 0.5U of *Taq*polymerase in a final volume of 25 μl. All the microsatellites were amplified by a BioRADthermal cycler at the following conditions: initial denaturation of 1 min at 95 °C, 30 cycles of 1 min at 95 °C, 1 min at T°C (optimum annealing temperature of each primer) and 1 min at 72 °C and a final extension of 5 min at 72 °C. Amplified fragments were separated by capillary electrophoresis using an ABI PRISM 3100 automatic sequencer (Applied Biosystems, Foster City, CA, USA) and allele sizing was accomplished by using the internal size standard GeneScan™-500LIZ™. Fluorescently labeled fragments were detected and sized using GeneMapper software (version 3.7, Applied Biosystems, USA). Stutter related scoring error, often seen in dinucleotide repeats, was absent and alleles could be scored unambiguously.

**Table 1:** Characteristics of 21 microsatellite loci used in present study

Primers	Primer sequences (5′-3′)	Forward label	Set	Annealing temperature	Product size (bp)	Total number of alleles
BM1824	F-gagcaaggtgtttttccaatc	VIC	4	58 °C	176-196	11
	R-cattctccaactgcttccttg					
CSSM08	F-cttggtgttactagccctggg	VIC	3	55 °C	182-200	8
	R-gatatatttgccagagattctgca					
CSSM33	F-cactgtgaatgcatgtgtgtgagc	NED	5	58 °C	144-188	21
	R-cccatgataagagtgcagatgact					
CSSM66	F-acacaaatcctttctgccagctga	FAM	4	60 °C	167-207	19
	R-aatttaatgcactgaggagcttgg					
ETH10	F-gttcaggactggccctgctaaca	NED	1	58 °C	185-221	14
	R-cctccagcccactttctcttctc					
ETH225	F-gaacctgcctctcctgcattgg	VIC	4	64 °C	134-162	13
	R-actctgcctgtggccaagtagg					
ETH3	F-gatcaccttgccactatttcct	NED	4	57 °C	90-124	16
	R-acatgacagccagctgctact					
HEL09	F-cccattcagtcttcagaggt	FAM	5	59 °C	140-182	17
	R-cacatccatgttctcaccac					
HEL5	F-gcaggatcacttgttaggga	VIC	3	55 °C	137-195	25
	R-agacgttagtgtacattaac					
ILSTS06	F-tgtctgtatttctgctgtgg	FAM	5	58 °C	275-303	14
	R-acacggaagcgatctaaacg					
ILSTS11	F-gcttgctacatggaaagtgc	NED	1	58 °C	249-273	10
	R-ctaaaatgcagagccctacc					
ILSTS34	F-aagggtctaagtccactggc	VIC	5	59 °C	138-212	37
	R-gacctggtttagcagagagc					
ILSTS33	F-tattagagtggctcagtgcc	PET	3	55 °C	131-163	16
	R-atgcagacagttttagaggg					
INRA05	F-caatctgcatgaagtataaatat	FAM	2	54 °C	130-148	9
	R-cttcaggcataccctacacc					
INRA35	F-atcctttgcagcctccacattg	FAM	3	54 °C	80-142	24
	R-ttgtgctttatgacactatccg					
INRA63	F-atttgcacaagctaaatctaacc	PET	2	54 °C	162-190	14
	R-aaaccacagaaatgcttggaag					
MM12	F-caagacaggtgtttcaatct	PET	4	52 °C	88-134	21
	R-atcgactctggggatgatgt					
MM8	F-cccaaggacagaaaagact	NED	2	55 °C	114-144	12
	R-ctcaagataagaccacacc					
TGLA122	F-ccctcctccaggtaaatcagc	VIC	1	58 °C	133-179	20
	R-aatcacatggcaaataagtacatac					
TGLA227	F-cgaattccaaatctgttaatttgct	PET	2	55 °C	67-119	17
	R-acagacagaaactcaatgaaagca					
TGLA53	F-gctttcagaaatagtttgcattca	FAM	1	58 °C	142-184	21
	R-atcttcacatgatattacagcaga					

### Microsatellite statistical analysis

GENALEX 6.2 software [15] was used to estimate basic population genetic descriptive statistics for each marker and population: gene frequency, observed number of alleles (No), number of private alleles, effective number of alleles (Ne), observed (Ho) and expected heterozygosity (He) and Analysis of Molecular Variance (AMOVA). The distribution of genetic variability between various breeds was studied by analyzing the Wright’s *F*-statistics (F_IS_ (f), F_ST_ (θ) and F_IT_ (F) and Nei’s [16] standard genetic distances among populations. Pair wise matrix of the genetic distances was then used to obtain Neighbor-joining (NJ) tree which was visualized using the software TreeView [17]. Bootstraps of 1000 replicates were performed in order to test the robustness of tree topology using the Phylip software [18]. The software GENEPOP version 3.4 [19] was used to perform global and per locus/ per population Hardy-Weinberg equilibrium (HWE) test, and to test for genotypic linkage disequilibrium (LD). Markov Chain method was employed with 1000 dememorization steps, 100 batches and 10,000 iterations. An alternative model based on Bayesian clustering analysis was used to infer how many clusters or sub-populations (K) were most appropriate for interpreting the data without prior information on the number of locations at which the individuals were sampled as implemented in STRUCTURE v2.2 [20]. Simulation was performed using a burn-in period of 50,000 rounds followed by 30,000 MCMC (Marcov Chain Monte Carlo) iterations. Independent runs of K were performed from 1 to 15 clusters and were repeated five times to check the consistency of the results. To choose the optimal K, posterior probability was calculated for each value of K using the mean estimated log-likelihood of K, L(K). Following Evanno et al. [21], delta K was calculated for each tested value of K (except for the maximum K tested), which is an ad-hoc statistic that is based on the second derivative of ‘the likelihood function with respect to K, L” (K). Graphic representation of these statistics was obtained using the web-based STRUCTURE Harvester software [22]. Principal Coordinate Analysis (PCoA) was employed for deciphering the population structure as implemented in GENALEX 6.2 software [15] and Principal Component Analysis (PCA) by XLSTAT version 2015.1.03.16133; Copyright Addinsoft 1995-2014 software.

### Mitochondrial DNA sequencing

The non-coding D-loop region was amplified by PCR, using primer pair (5΄-TAGTGCTAATACCAACGGCC-3΄, 5΄-AGGCATTTTCAGTGCCTTGC-3΄), as described by Suzuki et al. [23]. The D-loop primers yielded a PCR product of 1142 bp representing the whole D-loop and flanking sequence at both ends. Polymerase Chain Reaction (PCR) was carried out on about 50-100 ng genomic DNA in a 25 μl reaction volume using i-cycler (BioRAD, USA). The reaction mixture consisted of 200 μM of each dNTPs, 1.5 mM MgCl_2_, 50pmol primer, 0.5 U *Taq* polymerase (Bangalore GeneiPvt Ltd., Bangalore, India) and *Taq* buffer. Negative controls (lacking template DNA) were included in all reactions, and produced no products. The PCR reaction cycle was accomplished by denaturation for 6 min at 94 °C, 30 cycles of 94 °C for 45 s, 60 °C for 30 s, 72 °C for 60 s, and finally extension at 72 °C for 6 min, before cooling to 4 °C for 10 min. The size of amplification product was checked by loading 5 μL PCR product ontoa 1.8 % agarose gel containing 0.5 μL/mL ethidium bromide. The product was purified usinga QIA quick PCR purification kit (Qiagen, Hilden, Germany). Purified product was labeledusing the BigDye Terminator 3.1 Cycle sequencing kit (Applied Biosystems, Foster City, CA,USA) and sequenced directly using an ABI3100 Prism automatic DNA sequencer followingmanufacturer instructions. The primers used for sequencing were the same as those used in the PCR. Both strands of PCR product were completely sequenced. All finalsequences were determined from both strands for verification.

### Mitochondrial DNA statistical analysis

The DNA sequences were edited manually using EDITSEQ (DNASTAR) and the MegAlign program (DNASTAR) was used for multiple alignments. Sites representing a gap in any of the aligned sequences were excluded from the analysis. We compared 60 D-loop haplotypes of a 230-bp hypervariable region-I (HVR-I) fragment of mtDNA control region obtained from 170 cattle from India. Mean number of pairwise differences and nucleotide diversity (π) within cattle breeds, nucleotide divergence between breeds and haplotype diversity (H_d_) of breeds were calculated by Arlequin 3.1 [24]. The Neighbour-joining treebased on the HYR-I sequences was reconstructed using MEGA software [25]. Network analysis was used to visualize the spatial distribution of the sequence variation among the different mtDNA haplotypes. Network profiles among haplotypes were constructed by median-joining networks (NETWORK 4.5; http://www.fluxus-engineering.com/sharenet.htm), resolving the reticulations through a maximum parsimony criterion [26].

## Results

### Microsatellite and Mitochondrial genetic variability

Genetic status and diversity of indigenous cattle populations of India was established using nuclear (microsatellite markers) and mitochondrial polymorphisms. All microsatellite markers used in this study were successfully amplified in five multiplex sets designed with consideration for annealing temperature, product size and specific dye label in all the populations (Table 1). The genotype data generated in present study showed that significant amount of genetic variation is maintained in indicine cattle populations. All the markers were found to be polymorphic in each of the eleven populations analyzed. Considering all the populations, majority of the markers were in Hardy-Weinberg Equilibrium (HWE). Deviations from HWE were statistically significant (P < 0.01) in 5 (Bachaur, Gaolao), 4 (Ongole, Purnea, Kenkatha, Kherigarh), 3 (Hariana, Mewati, Ponwar, Shahabadi) and 2 (Gangatiri) loci. The level of variations depicted by number of alleles at each locus serves as a measure of genetic variability having direct effect on differentiation of breeds within a species [27]. Thus, FAO has specified a minimum of four different alleles per locus for evaluation of genetic differences between breeds. By this criterion, all the 21 microsatellite loci showed ample polymorphism for evaluating within breed genetic variability and exploring genetic differences between breeds as four or more alleles were observed at each loci.

A total of 359 alleles were detected with ILSTS34 presenting the highest number of alleles per locus (37) while CSSM08 was least (8 alleles) polymorphic. The average observed number of alleles per locus ranged from 6.571 ± 0.732 in Hariana to 10.619 ± 0.824 in Shahabadi cattle with the mean allele number across all the loci of 8.784 ± 0.25 (Table 2). The average effective number of alleles in a population varied from 3.374 ± 0.329 (Hariana) to 4.745 ± 0.532 (Shahabadi). Lower values of expected number of alleles as compared to observed number of alleles in all the populations suggested that there were many low frequency alleles in the populations. The private alleles, confined to one population only, ranged between none (Bachaur, Gangatiri, Kenkatha, Ponwar) and 24 (Ongole). Most of them were rare alleles with allele frequencies <5 % at each locus in each population. But still there were 24 private alleles at all loci across all populations with allele frequencies >5 %, and occurrence of these alleles can lead towards genetic signatures for a particular population. No significant linkage disequilibrium was detected between any two of these loci which were located on a single chromosome, and thus all were retained for diversity and differentiation analysis.

**Table 2:** Genetic diversity indices (Average) across 11 Indian cattle breeds with 21 microsatellite markers

Cattle population	Na	Ne	Ho	He	F_is_
Bachaur	9.476 ± 0.752	4.186 ± 0.440	0.694 ± 0.038	0.705 ± 0.030	0.017
Gangatiri	9.190 ± 0.716	4.117 ± 0.436	0.709 ± 0.034	0.702 ± 0.030	−0.010*
Kherigarh	9.238 ± 0.889	4.086 ± 0.444	0.704 ± 0.035	0.700 ± 0.029	−0.002
Kenkatha	9.000 ± 0.878	4.123 ± 0.409	0.724 ± 0.036	0.703 ± 0.030	−0.028*
Ponwar	8.857 ± 0.804	4.329 ± 0.518	0.696 ± 0.039	0.702 ± 0.031	0.014
Shahabadi	10.619 ± 0.824	4.745 ± 0.532	0.713 ± 0.035	0.735 ± 0.027	0.034*
Purnea	8.905 ± 0.771	4.072 ± 0.402	0.681 ± 0.040	0.706 ± 0.027	0.042*
Mewati	7.762 ± 0.730	3.451 ± 0.425	0.579 ± 0.049	0.634 ± 0.043	0.098*
Gaolao	9.143 ± 0.762	4.176 ± 0.383	0.616 ± 0.034	0.717 ± 0.026	0.146*
Hariana	6.571 ± 0.732	3.374 ± 0.329	0.604 ± 0.052	0.632 ± 0.049	0.042*
Ongole	7.667 ± 1.107	4.223 ± 0.698	0.459 ± 0.068	0.594 ± 0.078	0.221*
**Mean** ± **SE**	8.784 ± 0.252	4.082 ± 0.139	0.653 ± 0.014	0.685 ± 0.012	0.048 ± 0.017

Na- Observed number of alleles, Ne-Expected number of alleles, Ho-Observed heterozygosity; He-Expected heterozygosity, F_is_- Inbreeding coefficient, *(*p* <0.05)

Estimates of observed heterozygosity including all loci and populations (0.653 ± 0.01) confirmed the remarkable level of diversity in the Indian cattle. Among populations, observed heterozygosity ranged from 0.459 ± 0.07 to 0.724 ± 0.036 with the lowest value found in Ongole cattle and the highest in Kenkatha cattle (Table 2). Observed heterozygosity was lower than the expected heterozygosity in Bachaur, Ponwar, Shahabadi, Purnea, Mewati, Gaolao, Hariana and Ongole cattle populations. Analysis of F_IS_ evidenced heterozygote deficiency which was highest in Ongole (22.1 %) and lowest in Ponwar (1.4 %).

A fragment of 230 bp hypervariable region-I (HVR-I) of the non-coding mtDNA control region was unambiguously explored resulting in identification of 223 variable sites. Consequently, 60 haplotypes were identified with haplotypic diversity of 0.90240 (Table 3). The mtDNA control region haplotype sequences were deposited in GenBank [KP223257– KP223282]. An overall estimate for population indices revealed nucleotide diversity of 0.02688 and average number of nucleotide differences as 6.07407. These indices indicated sufficient mtDNA diversity amongst the analyzed breeds. Haplotype diversity (H_d_) was high in all the populations, ranging from 0.80526 (Hariana) to 0.96429 (Ponwar).

**Table 3:** Variability of the mtDNA control region sequences of Indian cattle

Cattle population	Number of sequences	Number of segregating sites	Number of haplotypes	Haplotype diversity, H_d_	Average number of differences	Nucleotide diversity, π
Bachaur	9	10	6	0.88889	2.55556	0.01131
Kenkatha	6	25	4	0.86667	8.60000	0.03805
Kherigarh	14	31	10	0.93407	5.96703	0.02640
Ponwar	8	13	7	0.96429	3.96429	0.01754
Purnea	26	38	20	0.95077	4.56308	0.02019
Shahabadi	31	33	15	0.89032	4.65806	0.02061
Gaolao	9	7	5	0.80556	2.38889	0.01057
Hariana	20	9	7	0.80526	2.14211	0.00948
Mewati	22	29	12	0.89610	4.89177	0.02165
Ongole	25	28	10	0.82000	12.10667	0.05357

### Population differentiation

Results of F-statistics for each of the 21 loci across populations are presented in Table 4. The global deficit of heterozygotes across populations (F_IT_) amounted to 17.5 % (*P* <0.001). An overall significant deficit of heterozygotes (F_IS_) of 4.9 % occurred in the analyzed loci because of inbreeding within populations. The multi-locus F_ST_ values of breed differentiation indicated that 13.3 % of the total genetic variation was due to unique allelic differences between the breeds, with the remaining 86.7 % corresponding to differences among individuals within the breed across the 21 markers. All loci contributed to the differentiation with the highest values found for ETH225 (32.4 %). The pair-wise F_ST_ values of breeds (Table 5) ranged between 0.007 to 0.261, thereby revealing the least differentiation between Ponwar-Kenkatha (0.007), Bachaur-Gangatiri, Bachaur-Kenkatha (0.008), Bachaur-Kherigarh, Gangatiri-Kenkatha, Kherigarh-Kenkatha, Kherigarh-Ponwar (0.009) and the highest divergence between Ongole and all other breeds of Northern India (>0.2). Similarly, AMOVA revealed that percent of variation among the populations was 24 % while within the population it was 76 %.

**Table 4:** Global *F*-Statistics for each of 21 microsatellite loci analyzed across 11 cattle populations

Locus	*Fis*	*Fit*	*Fst*	Nm
BM1824	0.144	0.188	0.051	4.632
CSSM08	0.025	0.143	0.121	1.820
CSSM33	0.011	0.076	0.067	3.508
CSSM66	0.018	0.052	0.035	6.851
ETH10	−0.111	−0.063	0.043	5.608
ETH225	0.014	0.333	0.324	0.522
ETH3	−0.121	0.094	0.192	1.051
HEL09	−0.037	0.117	0.149	1.427
HEL5	0.121	0.250	0.147	1.447
ILSTS06	0.153	0.223	0.084	2.743
ILSTS11	0.185	0.359	0.214	0.919
ILSTS34	0.096	0.167	0.078	2.950
ILSTS33	0.075	0.155	0.086	2.661
INRA05	0.019	0.168	0.152	1.395
INRA35	0.042	0.235	0.201	0.994
INRA63	0.054	0.208	0.163	1.284
MM12	0.087	0.117	0.033	7.359
MM8	0.083	0.262	0.195	1.030
TGLA122	0.091	0.120	0.032	7.532
TGLA227	0.060	0.341	0.299	0.586
TGLA53	0.013	0.131	0.119	1.848
Mean ± SE	0.049 **±** 0.017	0.175 **±** 0.022	0.133 **±** 0.018	2.770 **±** 0.498

**Table 5:** *Fst* estimates between each pair of eleven Indian cattle populations

Bachaur	Gangatiri	Kherigarh	Kenkatha	Ponwar	Shahabadi	Purnea	Mewati	Gaolao	Hariana	Ongole	
0.000											Bachaur
0.008	0.000										Gangatiri
0.009	0.010	0.000									Kherigarh
0.008	0.009	0.009	0.000								Kenkatha
0.010	0.012	0.009	0.007	0.000							Ponwar
0.032	0.031	0.032	0.034	0.032	0.000						Shahabadi
0.032	0.033	0.032	0.032	0.031	0.033	0.000					Purnea
0.101	0.101	0.101	0.091	0.097	0.081	0.094	0.000				Mewati
0.052	0.057	0.054	0.050	0.051	0.042	0.050	0.062	0.000			Gaolao
0.105	0.106	0.106	0.102	0.106	0.091	0.102	0.087	0.068	0.000		Hariana
0.212	0.210	0.213	0.213	0.212	0.203	0.206	0.257	0.201	0.261	0.000	Ongole

**Fig. 2:**
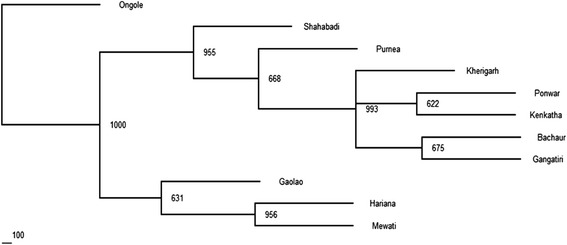
Dendrogram (NJ) showing genetic relationships among eleven Indian cattle populations based on Nei’s distance. The numbers at the nodes are bootstrap values from 1,000 replications

**Fig. 3:**
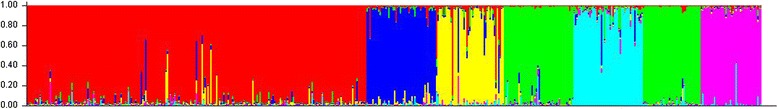
Clustering assignment of 508 animals representing eleven Indian cattle populations using STRUCTURE at K = 6. Each individual cattle is represented as a thin vertical line that is divided into segments whose size and color correspond to the relative proportion of the animal genome corresponding to a particular cluster. Shahabadi (Royal Blue), Purnea (Yellow), Gaolao (Sky blue) and Ongole (Pink) form separate cluster. Ponwar, Kherigarh, Kenkatha, Bachaur and Gangatiri (Red) cluster in one group and Hariana and Mewati (Green) form one cluster

Visualization of breed relationship was done by constructing Neighbor joining tree on the basis of Nei’s genetic distance. As expected, the Ongole was most distinct and separated first, while remaining populations formed two groups with clustering of Hariana, Mewati and Gaolao on one node and all other north Indian breeds on second with more than 95 % bootstrap value (Fig. 2). This grouping pattern was further supported by Principal Coordinate Analysis (PCoA). First three dimensions of the PCoA (PC1 = 44.59; PC2 = 28.97; PC3 = 10.88) accounted for 84.44 % of total variation. Ongole was distinct from the rest of populations, Hariana and Mewati were closer and fall in a different quadrant along with Gaolao whereas, Kenkatha, Ponwar, Kherigarh, Gangatiri, Bachaur, Shahabadi and Purnea clustered together in one quadrant (Additional file 1: Figure S1). The results of the PCA are in concordance with the phylogenetic tree obtained in the present study (Additional file 1: Figure S1), with the first two components accounting for 92.47 % of the total variation among the populations. Likely value of K which best captures the variation present in the data following the Bayesian approach employed in software STRUCTURE was six based on modal value of K versus K distribution following Evano et al. [21]. Ongole, Gaolao, Purnea and Shahabadi were grouped in their own clusters. However, Hariana and Mewati animals partitioned into one cluster (Fig. 3). The results are coincident with genetic distance among the populations as divergence was lowest between Bachaur, Gangatiri, Kherigarh, Kenkatha and Ponwar (Additional file 2: Table S1). The assignment test based on likelihood method with the leave one out procedure [15] assigned 74 % of the individuals correctly to their respective populations. All the individuals of Mewati, Gaolao, Hariana and all except one of Ongole and Shahabadi were assigned correctly, exhibiting distinctiveness of these breeds (Additional file 3: Sheet S1).

**Fig. 4:**
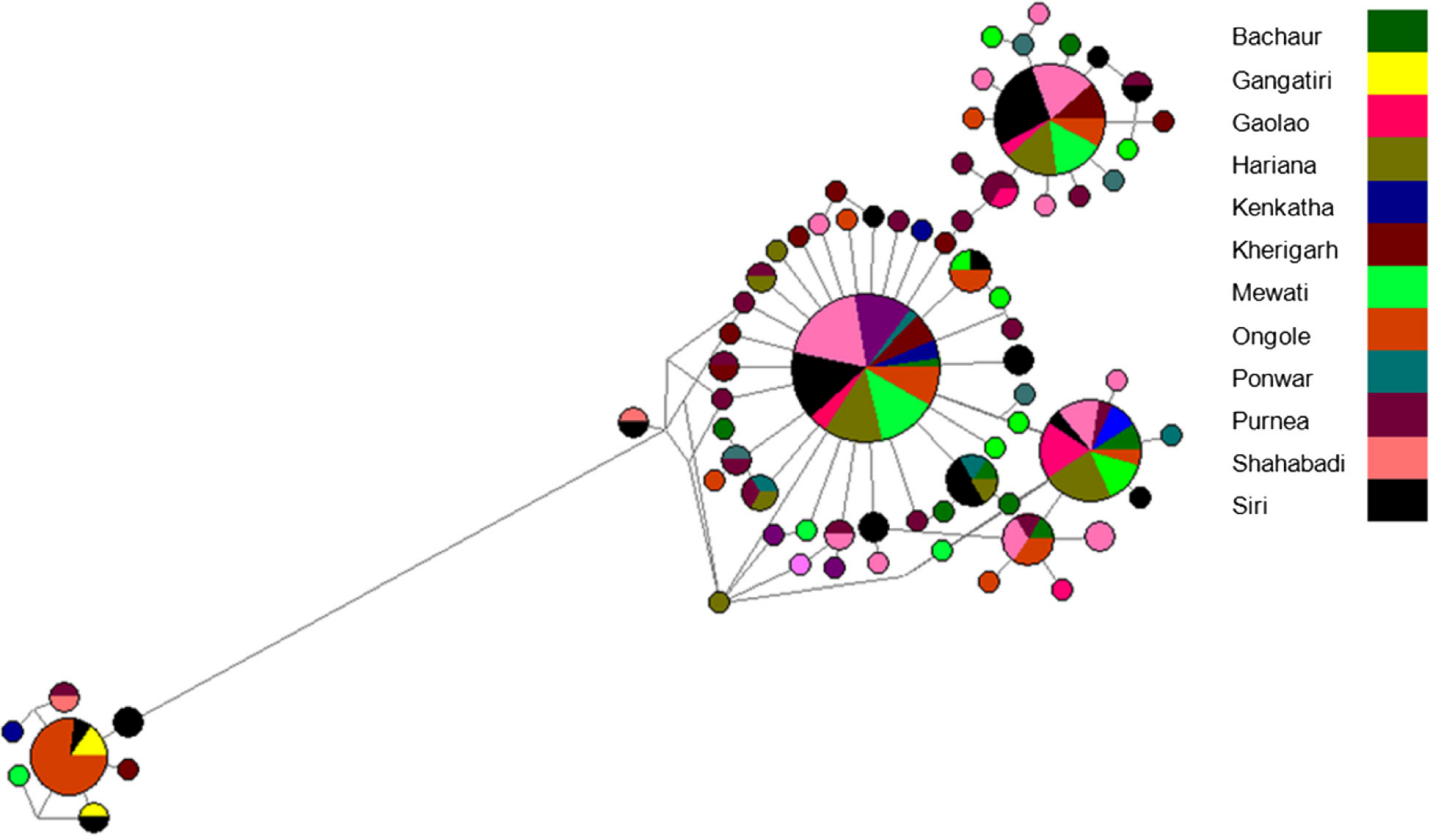
Median-Joining network of haplotypes belonging to 170 Indian autochthonous cattle analyzed in this study. The size of node is proportional to the haplotype frequency

The overall pair wise comparison of mismatch distribution of mitochondrial sequences revealed a predominant peak at around 1 mismatch (pairwise differences). However, a minor peak at 22 and 24 mismatches was also observed (Additional file 4: Figure S2). The individuals from major group differed from each other by 1 to 7 mismatches, while the individuals from minor group differed by 20 to 29 mismatches. Phylogenetic relationship based on mtDNA haplotype revealed the clustering of breeds in two major clades, according to their geographic locations (Additional file 5: Figure S3). The breeds form northern/central regions were phylogeographically separated from Ongole breed of Southern region. The mtDNA haplotype data was further utilized to generate network using median-joining algorithm. The median network exhibited a complex network for haplotypes with two major star clusters indicating population expansion for Indian cattle (Fig. 4). This demography of population expansion was in accordance with the mismatch distribution.

## Discussion

Molecular information is crucial for preserving genetic diversity as well as preventing undesirable loss of alleles. In this study genetic diversity and population structure of Indian cattle was estimated using nuclear and mitochondrial DNA polymorphism.

### Genetic diversity of Indian cattle

In general, genetic variation of the eleven populations is high according to the allele numbers and heterozygosity values of the microsatellite loci (Table 2) and the sequence divergence of mitochondrial hypervariable region-I (Table 3). The mean observed number of alleles across all the microsatellite loci were 8.784 ± 0.25 and were higher than other indigenous cattle breeds [28–30]. Lower allelic diversity than studied populations has also been reported in exotic cattle-Burlina-6.7 [31], Brown Swiss-5.4 [32] and Creole cattle-7.2 [33]. Previously also the allelic diversity in the Indian livestock breeds has been observed to be higher than that reported for the European counterpart [34]. This might be attributed to lack of artificial selection pressure and also indicates large effective population size of investigated Indian cattle populations. Allelic diversity of similar magnitude has also been reported in Tharparkar, Rathi and Orissa cattle populations of India [8, 12]. Measures of genetic diversity based on allelic richness are considered important in conservation genetics as marker-assisted methods for maximizing number of alleles conserved have been shown to be effective [35]. It is also relevant in long-term perspective, as selection limits are determined by the initial allelic composition rather than by heterozygosity [36].

Estimates of observed heterozygosity including all loci and population (0.653 ± 0.014) confirm the remarkable level of diversity in the studied populations. Higher genetic variation in Indian cattle must have contributed to its adaptability as genetic variation is necessary to allow organisms to adapt to ever changing environments with some of this variation stemming from introduction of new alleles by the random and natural process of mutation. Overall heterozygosity estimates were comparable with Tharparkar cattle (0.64) [8], Orissa cattle populations (0.62 to 0.66) [12] of India, Chinese cattle (0.62) [37] and Creole cattle (0.61) [33]. The least observed (0.459) and expected heterozygosity (0.594) values were detected for Ongole. The highest heterozygosity in Shahabadi population (0.735) could be explained by the occurrence of low selection pressure due to the lack of breeding programs. Similarly high mtDNA diversity as reflected in haplotypic (H_d_) and nucleotide diversity (π) is also congruent with previous results of Indian cattle [38, 39]. Higher genetic diversity of Indian cattle can be due to less emphasis on programmed breeding strategies. An additional source for increased indicine diversity could be the involvement of several species leading to admixture as suggested by Decker et al. [40] using genotypes from 43,043 autosomal single nucleotide polymorphism markers, scored in 1,543 animals involving high-throughput genotyping assays.

Significant heterozygote deficit (F_IS_) was observed for eight of the 12 breeds investigated being highest in Ongole (0.221). On the contrary, Kenkatha, Kherigarh and Gangatiri presented slight heterozygote excess in the population (-0.028, 0.002,-0.010, respectively) which was expressed in heterozygosity pattern too (Table 2). These results can be interpreted as possible signs of outbreeding, most likely due to recent admixture of two (or more) populations. Free grazing of these animals with the non-descript animals in a herd could be the likely source for the excess heterozygotes. Positive F_IS_ estimate for remaining populations indicates either the presence of inbreeding and /or Wahlund effect (presence of population substructure within breed). Since blood samples were collected from different villages, presence of a hidden substructure cannot be ruled out. Paucity of pure bulls as well as management seems to be the main reasons for heterozygote deficiency in these cattle. Moreover exotic/crossbred semen (Jersey and Holstein Friesian) is available in the breeding tracts whereas, local bull semen is usually unavailable to the owners. Together these two factors are resulting in the reduction of true to the breed type animals. In case of draft breeds, most of the males are used for carrying loads and agricultural operations. These males are castrated around the age of one year leading to their genetic death. With the modernization of agriculture and sub-division of land holdings, bullock power in Indian agriculture is losing its importance. Thus, with the diminishing demand for bullock power, the farmers are not adequately motivated to conserve these draft breeds.

### Differentiation between southern Indian and central and northern Indian populations

The clustering solutions of nuclear and mitochondrial DNA showed extensive sharing of diversity and absence of genetic substructure between the geographically proximal populations and breeds. Our results showed that Southern Indian cattle (Ongole) and Central and Northern Indian cattle have distinctive genotypes, both in nuclear (Figs. 2, 3 and Additional file 1: Figuire S1) and in mitochondrial genomes (Fig. 4 and Additional file 5: Figure S3).

The studied populations showed a moderate and significant genetic differentiation (F_ST_ = 0.133 ± 0.018). These results reflect that within-breed genetic variation is more (86.7 %) than between-breed (13.3 %) and this variation could be a valuable tool for genetic improvement and conservation of cattle populations of India. Genetic differentiation of similar magnitude has been reported in some other indigenous cattle [9]. However, much lower F_ST_ value has been reported among cattle breeds of Orissa and hill cattle of Kumaun (0.044) from India [12], as well as zebu cattle of Bangladesh [41]. While, several reports on exotic cattle (*Bos taurus*) viz. North European breeds F_ST_ = 0.107 [42], seven European cattle breeds F_ST_ =0.112 [43] and Swiss cattle F_ST_ = 0.090 [32] also depicted lower genetic differentiation than populations investigated in this study. The higher value of genetic differentiation in Indian cattle in this case may be attributed to the fact that the breeds are geographically well separated from each other being distributed in three different regions of India and the divergence is due to the reproductive isolation by distance. Similarly high genetic differentiation was observed by Mukesh et al. [28] with three Indian cattle which were far apart in distribution (Sahiwal, Deoni and Hariana). Furthermore, five lines of evidence suggest that Indian cattle breeds are differentiated. First, visualization of breed relationship using NJ tree obtained from Nei’s genetic distance shows clustering of breeds in conformity to the geographic location of populations (Fig. 2). Secondly, these observations were supported by the PCoA, which graphically illustrated differentiation of Ongole from rest of Indian cattle and further differentiation of Hariana, Mewati and Gaolao from the remaining cattle breeds of Northern India (Additional file 1: Figure S1). Thirdly, assignment test could correctly assign individuals of five breeds. Fourthly, an alternative Bayesian approach followed to delineate clusters of individuals on the basis of their genotypes at multiple loci employed in software STRUCTURE illustrated strong genetic structure of the cattle population of south (Ongole) with respect to other cattle breeds. Graphical methods are loosely connected to statistical procedures for the identification of homogeneous clusters of individuals. Whereas, Bayesian clustering methods allow for the assignment of individuals to groups based on their genetic similarity and provide information about the number of populations underlying the observed genetic diversity. Lastly, the mutational dynamics of mtDNA sequences enable the genetic relationships among haplotypes to be inferred and also confirmed uniqueness of Ongole cattle. In totality, all the approaches confirmed that Ongole from South India formed its own distinct cluster.

These different lines of evidence suggest that some degree of genomic divergence has occurred between Ongole and other cattle breeds of India. The genomes of modern cattle basically reflect the history of animal movements by migratory farmers out of the ancient centers of the cattle domestication. At the time of Neolithic transition, zebu cattle were considered to be the most abundant and important domestic livestock species in Southern Asia. Indus Valley is the major centre of domestication for Indian cattle (*Bos indicus*) [40, 44]. However previous studies on Indian cattle have also proposed independent domestication centres (Indus valley, Ganges and South India) for Indian zebu (*Bos indicus*) [38, 45]. In the current study too, the network constructed using median-joining algorithm exhibits two star like expansion events radiating from two ancestral nodes revealing distinct dichotomy between southern cattle (Ongole) and other Indian cattle encompassing large separation time. This demography is further supported by the mismatch distribution where two smooth, bimodal distributions were separated by a large time interval (Additional file 4: Figure S2). The analysis for Indian cattle mtDNA haplotypes indicates the distinctness among star clusters (major proportion from Northern/central region) and an ancestral node from southern region separated with large number of mutation events (Fig. 4). Overall, the Southern breed ‘Ongole’ was distinct with respect to breeds from Northern/ Central India. This is also in concordance with the phylogeography of the analyzed breeds. South India has also been proposed as another independent centre of domestication within south Asia, specifically for crops [46]. Moreover, the morphological differences between cattle depicted in the rock art of South India and in the iconography of Indus Valley civilizations have also lead to the suggestions that the South India was a secondary centre for zebu domestication [46]. Further, remains of wild aurochs (*Bos primigenius*) have been clearly identified from Banahalli, Karnataka (India) [46, 47].

The inferences obtained from nuclear (STRs) and mitochondrial (D-loop) markers are consistent and in agreement with geographical distribution and historical backgrounds. Both proved the clear genetic differentiation between southern and other Indian cattle breeds. However, clustering solutions of mitochondrial and nuclear DNA showed extensive sharing of diversity and absence of genetic substructure between the breeds and populations of a single geographic area. Further studies involving genome-wide approaches are apparently needed for further elucidation of differentiation.

## Conclusion

This study involves detailed analysis of the genetic diversity and differentiation of Indian cattle from different regions. It is vital to report that indigenous cattle populations of India retain high levels of genetic diversity based on the results from analysis of two genetic markers (microsatellites and mtDNA control region). Inbreeding was detected in some breeds, suggesting the need for appropriate measures to be taken to avoid the negative effects. The results presented here can be used to assist all stakeholders as breeds with wide range of genetic diversity are required in the future for generating transgressive variation for quantitative loci mapping and developing new genotypes for particular management systems and market needs.

## Additional files

Additional file 1: Figure S1. Two-dimensional plot of the Principal Coordinate Analysis (PCoA) and Principal Component Analysis (PCA), depicting relative position of eleven cattle populations. Additional file 2: Table S1. Nei’s genetic distance between each pair of eleven Indian cattle populations. Additional file 3:
**Sheet S1.** Assignment of individuals to their reference populations (sheet 1) and pairwise population assignment graphs (sheet2). Additional file 4: Figure S2. Mismatch distribution constructed using mtDNA sequences analyzed for Indian cattle (*Bos indicus*) breeds in the present study. Additional file 5: Figure S3. mtDNA haplotype based UPGMA tree depicting phylogenetic relationship among Indian cattle breeds.

## Abbreviations

DNA: Deoxyribonucleic acid
dNTPs: Deoxynucleotide triphosphates
EDTA: Ethylene diamine tetra acetic acid
FAO: Food and agriculture organization of the United Nations
H_d_: Haplotype diversity
He: Expected heterozygosity
Ho: Observed heterozygosity
HVR-I: Hypervariable region-I
HWE: Hardy-Weinberg equilibrium
ICAR: Indian council of agricultural research
ISAG: International society for animal genetics
MCMC: Marcov Chain Monte Carlo iterations
MgCl_2_: Magnesium chloride
Mitochondrial D loop: mitochondrial displacement loop
mtDNA: Mitochondrial DNA
NCBI: National centre for biotechnology information
Ne: Effective number of alleles
NJ: Neighbor-joining tree
No: Observed number of alleles
PCA: Principal component analysis
PCR: Polymerase chain reaction
SoW-AnGR: The state of the world’s animal genetic resources
STRs: Short tandem repeats
UPGMA: Unweighted pair group method with arithmetic mean
