# Supplementary figures and images for: Genetic diversity and relationship of Indian cattle inferred from microsatellite and mitochondrial DNA markers
Source: BMC Genet. 2015 Jun 30;16:73. doi: 10.1186/s12863-015-0221-0 (PMC4485874; doi:10.1186/s12863-015-0221-0)

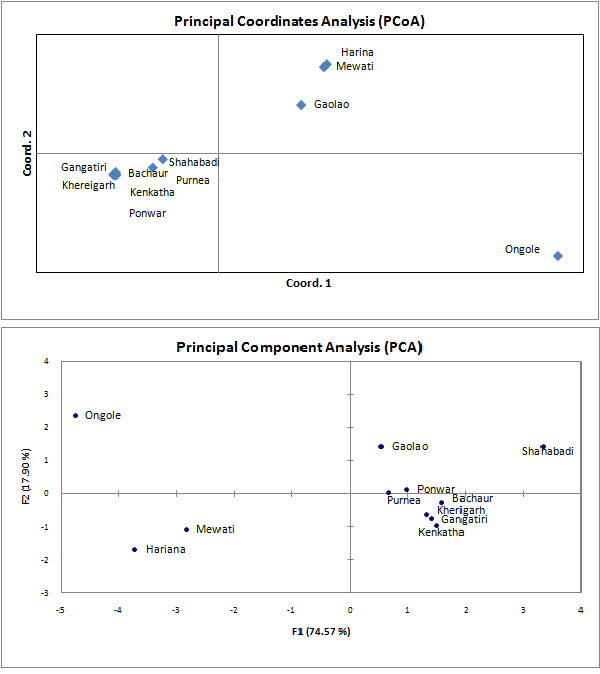

Supplement: Additional file 1: Figure S1. — Two-dimensional plot of the Principal Coordinate Analysis (PCoA) and Principal Component Analysis (PCA), depicting relative position of eleven cattle populations. [file 12863_2015_221_MOESM1_ESM.png]

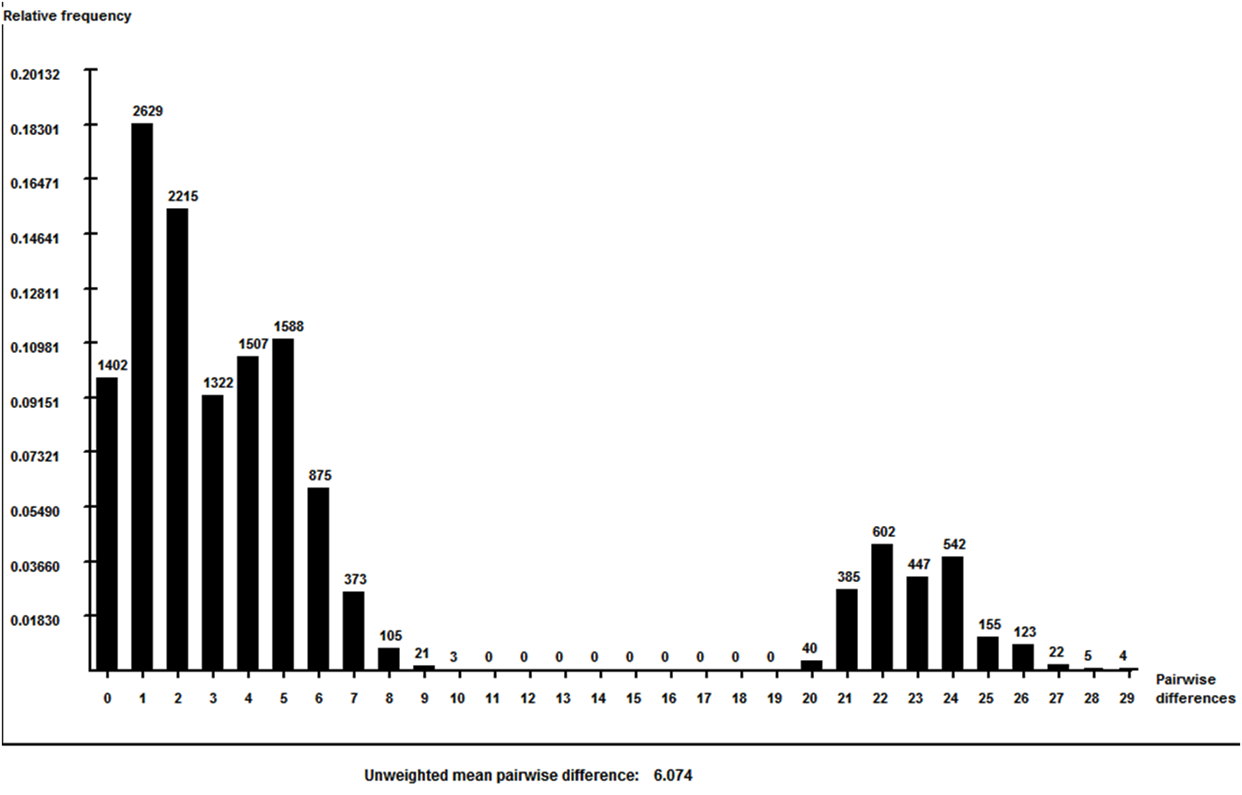

Supplement: Additional file 4: Figure S2. — Mismatch distribution constructed using mtDNA sequences analyzed for Indian cattle (Bos indicus) breeds in the present study. [file 12863_2015_221_MOESM4_ESM.png]

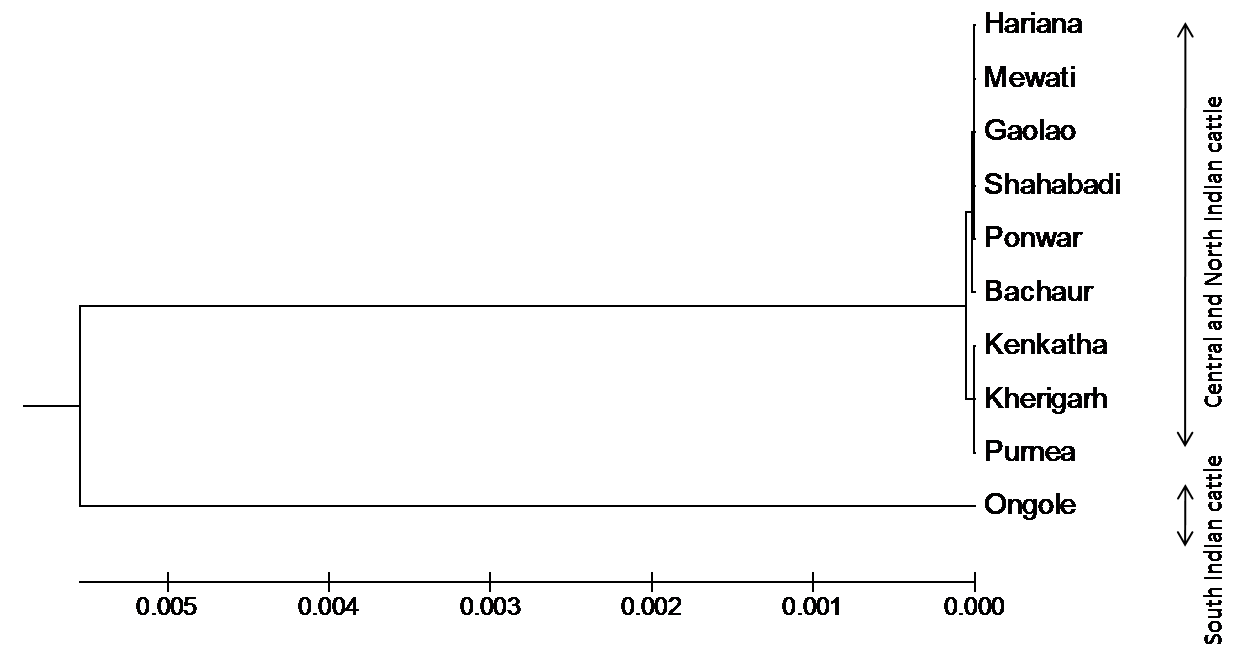

Supplement: Additional file 5: Figure S3. — mtDNA haplotype based UPGMA tree depicting phylogenetic relationship among Indian cattle breeds. [file 12863_2015_221_MOESM5_ESM.png]
