# Supplementary material for: Genetic diversity and relationship of Indian cattle inferred from microsatellite and mitochondrial DNA markers
Source: BMC Genet. 2015 Jun 30;16:73. doi: 10.1186/s12863-015-0221-0 (PMC4485874; doi:10.1186/s12863-015-0221-0)
Supplement: Additional file 2: Table S1. — Nei’s genetic distance between each pair of eleven Indian cattle populations. [file 12863_2015_221_MOESM2_ESM.docx]

**Table S1 Nei’s genetic distance between each pair of eleven Indian cattle populations**

|  | **Bachaur** | **Gangatiri** | **Kherigarh** | **Kenkatha** | **Ponwar** | **Shahabadi** | **Purnea** | **Mewati** | **Gaolao** | **Hariana** | **Ongole** |
| --- | --- | --- | --- | --- | --- | --- | --- | --- | --- | --- | --- |
| Bachaur | 0.000 |  |  |  |  |  |  |  |  |  |  |
| Gangatiri | 0.037 | 0.000 |  |  |  |  |  |  |  |  |  |
| Kherigarh | 0.044 | 0.048 | 0.000 |  |  |  |  |  |  |  |  |
| Kenkatha | 0.042 | 0.044 | 0.047 | 0.000 |  |  |  |  |  |  |  |
| Ponwar | 0.051 | 0.057 | 0.044 | 0.036 | 0.000 |  |  |  |  |  |  |
| Shahabadi | 0.191 | 0.183 | 0.189 | 0.199 | 0.190 | 0.000 |  |  |  |  |  |
| Purnea | 0.189 | 0.197 | 0.187 | 0.186 | 0.186 | 0.203 | 0.000 |  |  |  |  |
| Mewati | 0.782 | 0.781 | 0.774 | 0.740 | 0.784 | 0.641 | 0.730 | 0.000 |  |  |  |
| Gaolao | 0.631 | 0.665 | 0.653 | 0.620 | 0.640 | 0.574 | 0.593 | 0.199 | 0.000 |  |  |
| Hariana | 0.791 | 0.817 | 0.816 | 0.778 | 0.819 | 0.725 | 0.777 | 0.186 | 0.229 | 0.000 |  |
| Ongole | 1.075 | 1.052 | 1.101 | 1.109 | 1.076 | 1.092 | 0.967 | 1.071 | 0.695 | 1.110 | 0.000 |
